# Supplementary material for: Predictive Values of Nocturia and Its Voiding Frequency on the Aging Males’ Symptoms
Source: Int J Environ Res Public Health. 2022 Sep 15;19(18):11632. doi: 10.3390/ijerph191811632 (PMC9517266; doi:10.3390/ijerph191811632)
Supplement: Supplementary file 1 [file ijerph-19-11632-s001.zip › ijerph-1863158-supplementary.pdf]

Supplementary Table S1. The comparison between non-nocturia and nocturia on the severity levels of composite AMS and its subscales.

Variables	Total	Non-Nocturia	Nocturia	χ^2
	***N* = 1239**	***N* = 458**	***N* = 781**	**p-value**
		**Number (Percentage)**		
Composite AMS				<0.001
No/ Little	551 (44.5)	290 (63.3)	261 (33.4)	
Mild	444 (35.8)	136 (29.7)	308 (39.4)	
Moderate	222 (17.9)	30 (6.6)	192 (24.6)	
Severe	22 (1.8)	2 (0.4)	20 (2.6)	
Somato-vegetative				<0.001
No/ Little	129 (10.4)	90 (19.7)	39 (5.0)	
Mild	412 (33.2)	199 (43.5)	213 (27.3)	
Moderate	505 (40.8)	147 (32.1)	358 (45.8)	
Severe	193 (15.6)	22 (4.8)	171 (21.9)	
Psychological				<0.001
No/ Little	382 (30.8)	34 (41.3)	147 (24.7)	
Mild	562 (45.6)	98 (43.5)	350 (46.5)	
Moderate	242 (19.5)	30 (13.0)	123 (23.3)	
Severe	53 (4.2)	21 (2.2)	188 (5.5)	
Sexual				<0.001
No/ Little	310 (25.0)	34 (43.7)	147 (14.1)	
Mild	316 (25.5)	98 (26.9)	350 (24.7)	
Moderate	341 (27.5)	30 (18.6)	123 (32.8)	
Severe	272 (22.0)	21 (10.9)	188 (28.4)	

Supplementary Table S2. The comparison between non-nocturia and nocturia on the severity levels of 7 PSQI components.

Variables	Total	Non-Nocturia	Nocturia	χ^2
	N = 855	*N* = 344	*N* = 511	p-value
		Number (Percentage)		
Subjective sleep quality				<0.001
Very good	57 (6.7)	44 (12.8)	13 (2.5)	
Fairly good	556 (65.0)	249 (72.4)	307 (60.1)	
Fairly bad	217 (25.4)	49 (14.2)	168 (32.9)	
Very bad	25 (2.9)	2 (0.6)	23 (4.5)	
Sleep latency				<0.001
No difficulty	245 (28.6)	139 (40.4)	106 (20.7)	
Some difficulty	413 (48.3)	158 (46.2)	254 (49.7)	
More difficulty	156 (18.3)	41 (11.9)	115 (22.5)	
Severe difficulty	41 (4.8)	5 (1.5)	36 (7.1)	
Sleep duration				0.077
No difficulty	182 (21.3)	80 (23.3)	102 (20.0)	
Some difficulty	304 (35.6)	131 (38.1)	173 (33.8)	
More difficulty	269 (31.4)	103 (29.9)	166 (32.5)	
Severe difficulty	100 (11.7)	30 (8.7)	70 (13.7)	
Habitual sleep efficiency				<0.001
> 85%	572 (66.9)	281 (81.7)	291 (56.9)	
74 – 84%	170 (19.9)	46 (13.4)	124 (24.3)	
65 – 74%	89 (10.4)	15 (4.3)	74 (14.5)	
< 65%	24 (2.8)	2 (0.6)	22 (4.3)	

Sleep disturbance				<0.001
No difficulty	132 (15.5)	100 (29.1)	32 (6.3)	
Some difficulty	656 (76.7)	235 (68.3)	421 (82.4)	
More difficulty	65 (7.6)	9 (2.6)	56 (10.9)	
Severe difficulty	2 (0.2)	0 (0)	2 (0.4)	
Use of sleeping medication				<0.001
None	796 (93.1)	335 (97.4)	461 (90.2)	
< 1 per week	29 (3.4)	2 (2.0)	22 (4.3)	
1-2 per week	18 (2.1)	1 (0.3)	17 (3.3)	
≥ 3 per week	12 (1.4)	1 (0.3)	11 (2.2)	
Daytime dysfunction				0.069
No difficulty	401 (46.9)	161 (46.8)	240 (47.0)	
Some difficulty	303 (35.4)	134 (39.0)	169 (33.1)	
More difficulty	128 (15.0)	44 (12.8)	84 (16.4)	
Severe difficulty	23 (2.7)	5 (1.6)	18 (3.5)	

Supplementary Table S3. Severity levels of the 7 PSQI components associated with bedtime voiding frequency.

Variables	Episodes of nocturia				χ^2
	**1 Time**	**2 Times**	**3 Times**	**≥4 Times**	**p-value**
	N = 287	*N = 156*	*N = 47*	*N = 22*	
	**Number (Percentage)**				
Subjective sleep quality					<0.001
Very good	9 (1.1)	2 (1.3)	1 (2.1)	2 (9.1)	
Fairly good	193 (23.4)	74 (47.4)	19 (40.4)	11 (50.0)	
Fairly bad	74 (9.0)	74 (47.4)	24 (51.1)	6 (27.3)	
Very bad	11 (1.3)	6 (3.8)	3 (6.4)	3 (13.6)	
Sleep latency					<0.001
No difficulty	58 (7.0)	28 (17.9)	8 (17.0)	4 (18.2)	
Some difficulty	158 (19.2)	75 (48.1)	15 (31.9)	6 (27.3)	
More difficulty	62 (7.5)	41 (26.3)	13 (27.7)	6 (27.3)	
Severe difficulty	9 (1.1)	12 (7.7)	11 (23.4)	6 (27.3)	
Sleep duration					0.009
No difficulty	56 (6.8)	26 (16.7)	8 (17.0)	9 (40.9)	
Some difficulty	100 (12.1)	56 (35.9)	10 (21.3)	6 (27.3)	
More difficulty	100 (12.1)	51 (32.7)	18 (38.3)	3 (13.6)	
Severe difficulty	31 (3.8)	23 (14.7)	11 (23.4)	4 (18.2)	
Habitual sleep efficiency					<0.001
> 85%	173 (21.0)	81 (51.9)	17 (36.2)	12 (54.5)	
74 – 84%	78 (9.5)	41 (26.3)	10 (21.3)	2 (9.1)	
65 – 74%	31 (3.8)	30 (19.2)	12 (25.5)	4 (18.2)	
< 65%	5 (0.6)	4 (2.6)	8 (17.0)	4 (18.2)	
Sleep disturbance					<0.001
No difficulty	16 (1.9)	11 (7.1)	1 (2.1)	1 (4.5)	
Some difficulty	251 (30.5)	122 (78.2)	35 (74.5)	15 (68.2)	
More difficulty	20 (2.4)	21 (13.5)	11 (23.4)	6 (27.3)	
Severe difficulty	0 (0)	2 (1.3)	0 (00	0 (0)	
Use of sleeping medication					<0.001
None	268 (32.5)	140 (89.7)	35 (74.5)	20 (90.9)	
< 1 per week	11 (1.3)	7 (4.5)	4 (8.5)	0 (0)	
1-2 per week	4 (0.5)	7 (4.5)	3 (6.4)	2 (9.1)	

≥ 3 per week	43 (5.2)	2 (1.3)	5 (10.6)	0 (0)	
Daytime dysfunction					0.001
No difficulty	141 (17.1)	62 (39.7)	22 (46.8)	13 (59.1)	
Some difficulty	102 (12.4)	58 (37.2)	8 (17.0)	5 (22.7)	
More difficulty	40 (4.9)	28 (17.9)	13 (22.7)	2 (9.1)	
Severe difficulty	4 (0.5)	8 (5.1)	4 (8.5)	2 (9.1)	
